# 5-Amino-1-(2′,3′-*O*-iso­propyl­idene-d-ribit­yl)-1*H*-imidazole-4-carboxamide: a crystal structure with *Z*′ = 4

**DOI:** 10.1107/S2056989017000500

**Published:** 2017-01-13

**Authors:** Vincenzo Piccialli, Nicola Borbone, Giorgia Oliviero, Gennaro Piccialli, Stefano D’Errico, Roberto Centore

**Affiliations:** a Dipartimento di Scienze Chimiche, Università degli Studi di Napoli ‘Federico II’, Complesso di Monte S. Angelo, Via Cinthia, 80126 Napoli, Italy; b Dipartimento di Farmacia, Università degli Studi di Napoli ‘Federico II’, Via D. Montesano 49, 80131 Napoli, Italy

**Keywords:** crystal structure, high *Z*′ structures, nucleosides, hydrogen bonding

## Abstract

The title compound, C_12_H_20_N_4_O_5_, crystallizes in the monoclinic space group *P*2_1_, with four crystallographically independent mol­ecules in the asymmetric unit. The four mol­ecules have a very similar conformation that is basically determined by the formation of two intra­molecular hydrogen bonds between the amino NH_2_ donors and the carbonyl and ring O-atom acceptors, forming, respectively, *R*(6) and *R*(7) ring motifs.. In the crystal, inter­molecular hydrogen bonding leads to the formation of *R*_2_^2^(10) ring patterns, involving one amide CONH_2_ donor and an imidazole N-atom acceptor. The cluster of the four independent mol­ecules has approximate non-crystallographic *C*_2_ point symmetry. The structural analysis also shows that during the synthesis of the title compound, the reductive cleavage of the d-ribose ring of the inosine precursor proceeds stereoselectively, with retention of configuration.

## Abstract 2

The title compound crystallizes in the monoclinic space group *P*2_1_, with four crystallographically independent mol­ecules, having a very similar conformation, in the asymmetric unit. The cluster of independent mol­ecules has approximate non-crystallographic *C*_2_ point symmetry.

## Chemical context   

Our group has long been involved into the synthesis of new heterocyclic compounds (Piccialli *et al.*, 2007 ▸, 2013 ▸; Centore *et al.*, 2013 ▸) including novel bioactive nucleoside and nucleotide analogues (Galeone *et al.*, 2002 ▸). The latter are synthetic compounds that have been developed to mimic their natural counterparts (Jordheim, *et al.*, 2013 ▸). Several nucleoside and nucleotide analogues have been approved by the FDA for viral and cancer diseases and others have entered clinical trials. Therefore, the synthesis of new nucleoside analogues with potential biological activities (D’Atri *et al.*, 2012 ▸) continues to be a keen research field. Recent efforts from our group in this field have been directed to the synthesis of sugar and/or base-modified nucleosides (D’Errico *et al.*, 2012*a* ▸; de Champdorè *et al.*, 2004 ▸) and nucleotides, mixing the principles of combinatorial chemistry with those of high-throughput screening. Within this framework, we have pioneered the development of a synthetic solid-phase strategy (Oliviero *et al.*, 2007 ▸, 2008 ▸, 2010*a* ▸,*b* ▸; D’Errico *et al.*, 2011 ▸, 2015 ▸) that has also allowed us to synthesize *N*-1 alkyl inosines and 5-amino­imidazole-4-carboxamide riboside (AICAR) analogues (D’Errico *et al.*, 2012*b* ▸), starting from cheap inosine. AICAR is a purine biosynthetic precursor that acts as a modulator of a number of biological properties. Once in the cells, AICAR is 5′-phospho­rylated to ZMP, a mimic of adenosine 5′-monophosphate (AMP). The direct binding of ZMP to an allosteric site of AMPK causes its phospho­rylation and activation by a cellular kinase, resulting in a series of important metabolic events, including the inhibition of the basal and insulin-stimulated glucose uptake, the inhibition of lipid synthesis and the activation of certain ATP-generating processes such as glycolysis and fatty acid oxidation. Nevertheless, AICAR is far from being a good drug lead-compound because it has a short half-life in cells and is not strictly specific for the AMPK enzyme. The discovery of the anti­viral activity of a­cyclo­vir and acyclic nucleoside phospho­nates has suggested that the replacement of the furan­ose ring with a hy­droxy­lated alkyl chain could induce new biological activities. Based on these precedents, we have recently reported the synthesis of a small collection of 5-amino­imidazole-4-carboxamides carrying a d-ribityl chain at the *N*1-imidazole position, including the title compound (D’Errico *et al.*, 2013 ▸).
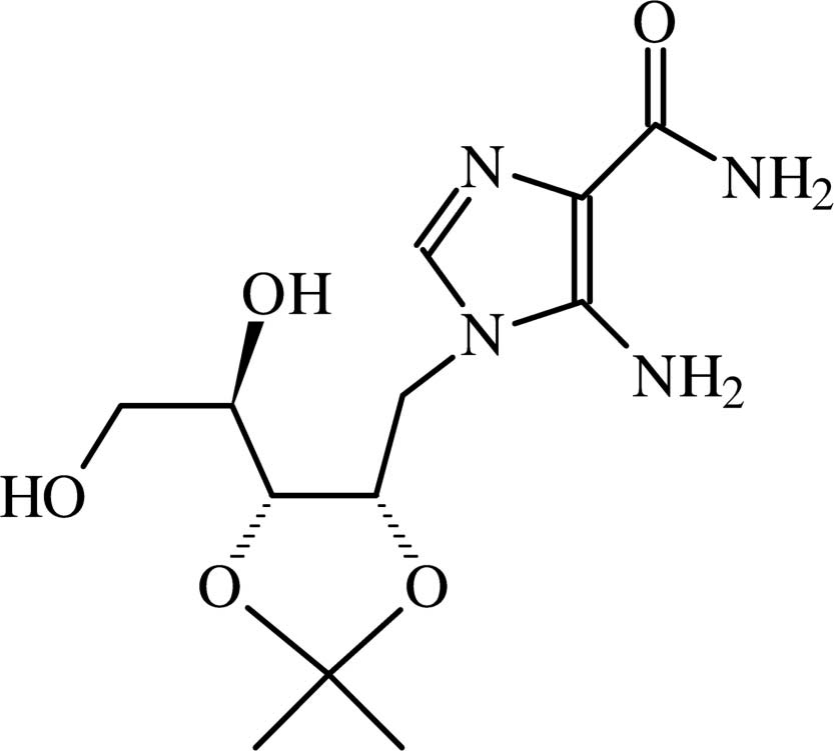


The present X-ray diffraction study was undertaken in order to confirm the structure of the title compound, 5-amino-1-(2′,3′-*O*-iso­propyl­idene-d-ribit­yl)-1-*H*-imidazole-4-carboxamide, a precursor of the new sugar-modified AICAR.

## Structural commentary   

**Figure 1:**
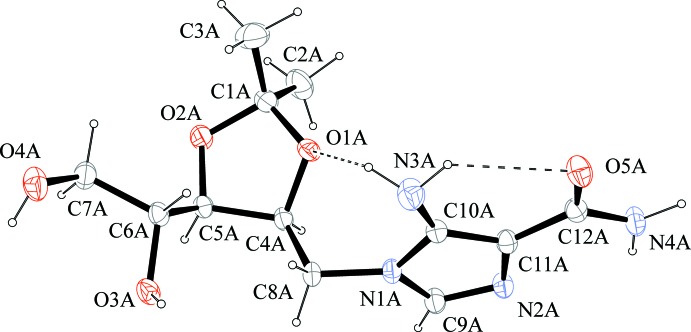
A view of the mol­ecular structure of one of the four crystallographically independent mol­ecules (*A*) of the title compound. Displacement ellipsoids are drawn at the 30% probability level. Intra­molecular hydrogen bonds are represented by dashed lines (see Table 1 ▸).

The asymmetric unit of the title compound contains four independent mol­ecules with identical configuration (Z′ = 4). The mol­ecular structure of one mol­ecule (*A*) is shown in Fig. 1 ▸ as an example. The mol­ecular conformation is basically determined by the formation of two intra­molecular hydrogen bonds (Table 1 ▸) between the amino NH_2_ donors and, respectively, the carbonyl (O5) and the ring (O1) acceptors, which form, respectively, *R*(6) and *R*(7) ring motifs. The formation of the intra­molecular hydrogen bonds is possible because of the pyramidal geometry of the N atom; the sums of valence angles around atoms N3*A*, N3*B*, N3*C* and N3*D* are, respectively, 336, 339, 334 and 337°.

**Figure 2:**
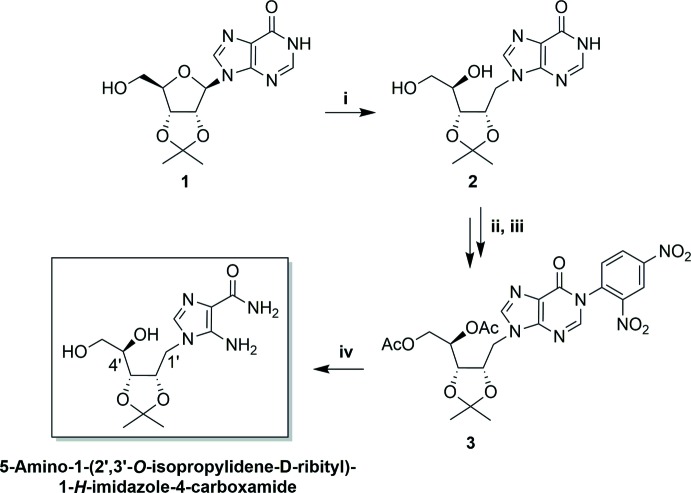
Scheme of the synthesis of the title compound. Reagents and conditions: (i) diiso­butyl­aluminium hydride (DIBAL-H), THF, 24 h, room temperature; (ii) Ac_2_O, py, 16 h, room temperature; (iii) K_2_CO_3_, 2,4-di­nitro­chloro­benzene (DNClB), DMF, 3 h, 353 K; (iv) Ethyl­enedi­amine (EDA), DMF, 323 K, 16 h.

The title compound was obtained starting from commercial 2′,3′-*O*-iso­propyl­idene inosine (compound **1** of Fig. 2 ▸) through a synthetic route involving four steps. In the first step [(i) of Fig. 2 ▸], the ribose ring is opened by reductive cleavage of the C1′—O4′ bond of 2′,3′-*O*-iso­propyl­idene inosine. The configuration of atom C4′ (C6*A* in Fig. 1) ▸ in the title compound is *R* and this confirms the stereoselectivity of the reductive ribose opening.

**Figure 3:**
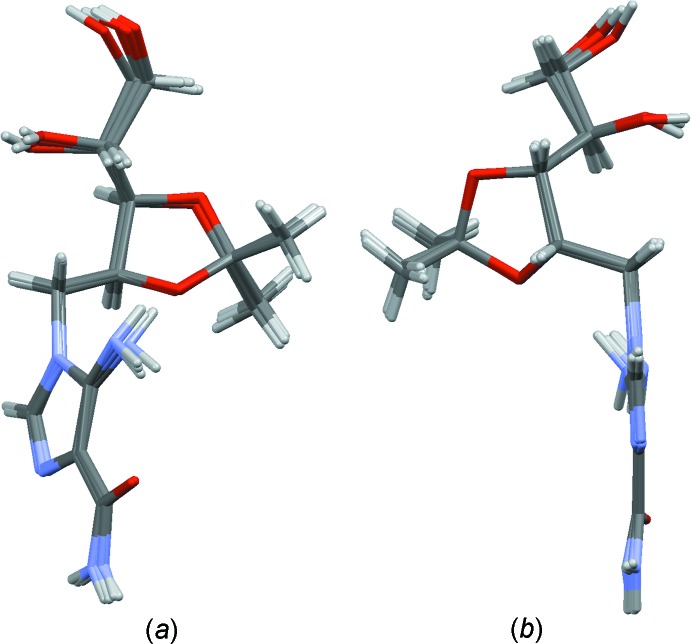
Overlay of the four crystallographically independent mol­ecules (*A*, *B*, *C* and *D*) of the title compound, viewed in two different orientations.

The four independent mol­ecules have a similar conformation. This can be inferred from Fig. 3 ▸, in which they are overlayed, and from Table 2 ▸ in which some parameters of the Hirshfeld surface of the four mol­ecules are presented (Spackman & McKinnon, 2002 ▸).

## Supra­molecular features   

**Figure 4:**
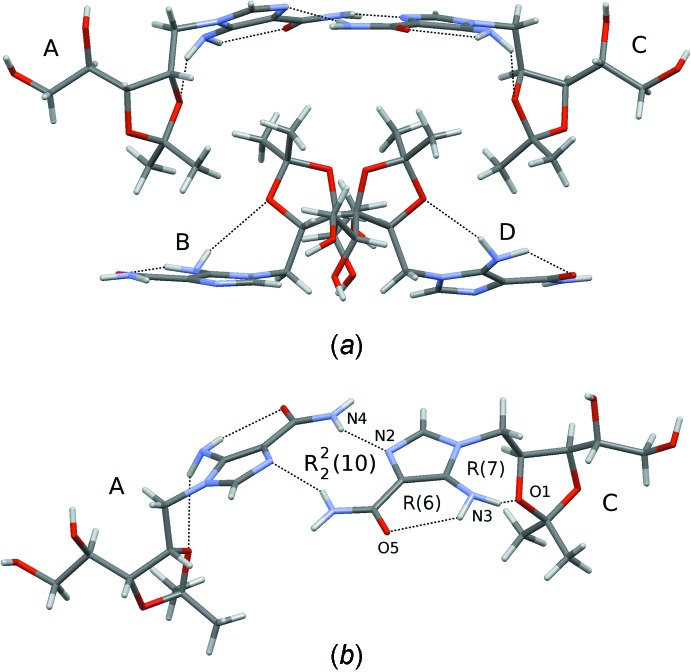
(*a*) The cluster formed by the four crystallographically independent mol­ecules (*A*, *B*, *C* and *D*) of the title compound. Hydrogen bonds are represented by dashed lines (see Table 1 ▸). (*b*) The pair of independent mol­ecules, *A* and *B*, with indication of some hydrogen-bonding patterns (dashed lines; see Table 1 ▸).

In the crystal of the title compound, the cluster of the four crystallographically independent mol­ecules (*A*, *B*, *C*, *D*) has approximate non-crystallographic *C*_2_ point symmetry, around a direction parallel to ***b***/2 + ***c***, see Fig. 4 ▸ *a*. Actually, the presence of non-crystallographic, local symmetry, is not uncommon in high *Z*′ structures (Brock, 2016 ▸). Mol­ecules are held in the crystal through a complex pattern of hydrogen bonds (Table 1 ▸). In particular, the independent mol­ecules *A* and *C* are hydrogen bonded through an 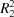
(10) ring pattern, involving one amido CONH_2_ donor and the imidazole N acceptor (Table 1 ▸ and Fig. 4 ▸ *b*). An analogous pattern is formed between mol­ecules *B* and *D*. As is evident from Fig. 4 ▸ *a*, in the cluster of four independent mol­ecules, the pair of mol­ecules (*A* and *C*) that are bonded through the 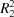
(10) ring pattern produce a hollow in which the methyl groups of the other pair (*B* and *D*) are fitted.

## Hirshfeld surface analysis   

**Figure 5:**
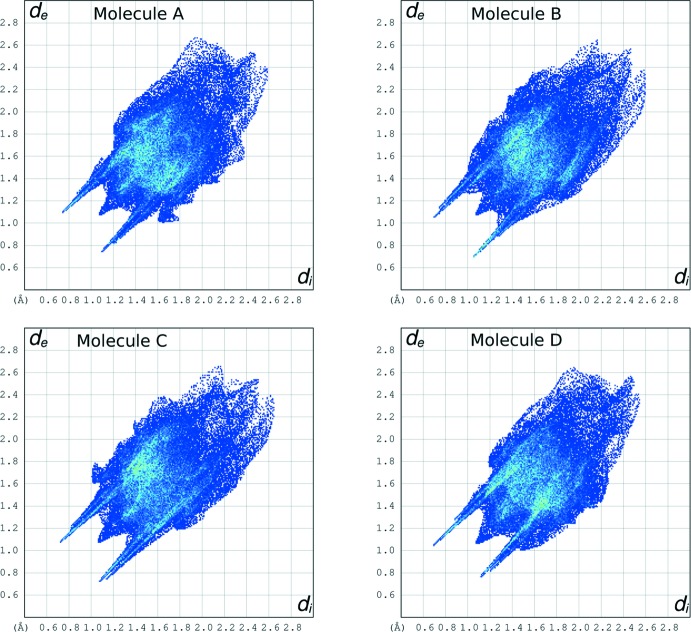
Hirshfeld fingerprint plots of the four crystallographically independent mol­ecules (*A*, *B*, *C* and *D*) of the title compound.

In order to assess possible packing differences involving the four independent mol­ecules, we have examined their Hirshfeld surfaces (Spackman & McKinnon, 2002 ▸). The Hirshfeld fingerprint plots of the four independent mol­ecules are illustrated in Fig. 5 ▸. The fingerprint plot is a graphical two-dimensional map that indicates the distribution of the inter­actions for a single mol­ecule in the crystal (Spackman & McKinnon, 2002 ▸). In the plot, for each point of the Hirshfeld surface enveloping the mol­ecule in the crystal, the distance *d_i_* to the nearest atom inside the surface and the distance *d_e_* to the nearest atom outside the surface are reported. The colour of each point in the plot is related to the abundance of that inter­action, from blue (low) to green (high) to red (very high). A distinctive feature of each plot of Fig. 5 ▸ is represented by the two spikes at *d_i_* + *d_e_* = 1.8 Å, pointing to the lower left of the plots and symmetrically disposed with respect to the diagonal. They correspond to the strong hydrogen bonds present in the crystal packing. Another common feature is the sting along the diagonal, at *d_i_* = *d_e_* = 1.05 Å, which reflects points on the Hirshfeld surface that involve nearly head-to-head H⋯H contacts. Although none of the four plots of Fig. 5 ▸ is superimposable on the others, they all look very similar, thus indicating that the packing around each mol­ecule is similar.

## Database survey   

A search of the Cambridge Structural Database (Groom *et al.*, 2016 ▸; WebCSD v1.1.2, last update 2016-12-21) gave no match for the title compound and no match for the substructure formed by the 1-amino-(2′,3′-*O*-iso­propyl­idene-d-ribit­yl) moiety. On the other hand, for the substructure formed by the uncyclized d-ribityl moiety, nine hits were found (CSD refcodes: ADRBFT10, DIQVAA, JERHET, QQQAVY, QQQHCA, RBFLAV10, RBFLCU, RIBBAD, RIBHQN10). They all crystallized in chiral space groups (four in *P*2_1_, three in *P*2_1_2_1_2_1_, one in *C*2 and one in *P*1). Only in two cases (both in space group *P*2_1_) was *Z*′ > 1 and, in particular, it was *Z*′ = 2. If the filters of three-dimensional coordinates and an *R* factor ≤ 7% are applied, only three hits still hold: DIQVAA (*P*2_1_), JERHET (*P*2_1_) and RBFLAV10 (*P*2_1_2_1_2_1_).

## Synthesis and crystallization   

The title compound, was synthesized starting from 2′,3′-*O*- iso­propyl­idene inosine (**1** in Fig. 1 ▸), as described recently (D’Errico *et al.*, 2013 ▸). In particular, compound **3** (0.18 mmol) was dissolved in DMF (2.0 ml) and then ethyl­ene di­amine (EDA, 3.6 mmol) was added. The mixture was stirred at 323 K for 16 h (TLC monitoring: CHCl_3_/MeOH, 8:2) and then the solvents were removed under reduced pressure. The crude product was purified by silica gel column chromatography, eluting with increasing amounts of MeOH in CHCl_3_ (from 0 to 10%). The fractions containing the title compound were collected and solvents evaporated under reduced pressure. The obtained pale-yellow amorphous solid (71% yield) was dissolved in the minimal amount of CH_3_OH and left to slowly evaporate at 277 K, to give pale-yellow prismatic crystals.

## Refinement   

Crystal data, data collection and structure refinement details are summarized in Table 3 ▸. The H atoms bonded to O and N atoms were located in difference Fourier maps and their coordinates were refined. The C-bound H atoms were included in calculated positions and refined as riding atoms: with C—H = 0.96–0.98 Å. For all H atoms, *U*_iso_ = 1.2*U*_eq_ of the carrier atom was assumed (1.5 in the case of the H atoms of methyl groups).

## Supplementary Material

Crystal structure: contains datablock(s) global, I. DOI: 10.1107/S2056989017000500/su5343sup1.cif

Structure factors: contains datablock(s) I. DOI: 10.1107/S2056989017000500/su5343Isup2.hkl

CCDC reference: 1526562

Additional supporting information:  crystallographic information; 3D view; checkCIF report

## Figures and Tables

**Table 1:** Hydrogen-bond geometry (Å, °)

*D*—H⋯*A*	*D*—H	H⋯*A*	*D*⋯*A*	*D*—H⋯*A*
N3*A*—H3*NA*⋯O5*A*	0.89 (4)	2.32 (4)	2.945 (4)	127 (3)
N3*A*—H6*NA*⋯O1*A*	0.89 (4)	2.66 (4)	3.250 (4)	125 (3)
N4*A*—H4*NA*⋯O3*A* ^i^	0.92 (4)	2.05 (4)	2.973 (3)	178 (3)
N4*A*—H5*NA*⋯N2*C*	0.91 (4)	2.23 (4)	3.064 (4)	153 (3)
O3*A*—H3*AO*⋯O4*C* ^ii^	0.79 (4)	2.05 (4)	2.831 (3)	170 (4)
O4*A*—H4*AO*⋯O5*A* ^iii^	0.80 (4)	2.01 (4)	2.798 (3)	166 (4)
N3*B*—H3*NB*⋯O1*B*	0.89 (3)	2.35 (3)	3.037 (3)	133 (3)
N3*B*—H6*NB*⋯O5*B*	0.95 (4)	2.39 (3)	2.977 (3)	120 (3)
N3*B*—H6*NB*⋯O5*D* ^iv^	0.95 (4)	2.52 (4)	3.196 (4)	129 (3)
N4*B*—H4*NB*⋯O3*B* ^iii^	0.89 (4)	2.03 (4)	2.901 (3)	170 (3)
N4*B*—H5*NB*⋯N2*D* ^iii^	0.85 (4)	2.16 (4)	2.920 (4)	150 (3)
O3*B*—H3*BO*⋯N3*C* ^v^	0.87 (3)	1.98 (4)	2.838 (4)	168 (3)
O4*B*—H4*BO*⋯O5*B* ^i^	0.91 (4)	1.81 (4)	2.715 (3)	170 (3)
N3*C*—H3*NC*⋯O1*C*	0.87 (4)	2.48 (4)	3.152 (4)	134 (3)
N3*C*—H6*NC*⋯O5*C*	0.90 (4)	2.21 (3)	2.874 (3)	130 (3)
N4*C*—H4*NC*⋯N2*A*	0.86 (4)	2.14 (4)	2.934 (4)	154 (3)
N4*C*—H5*NC*⋯O3*C* ^iii^	0.84 (4)	2.12 (4)	2.959 (3)	176 (4)
O3*C*—H3*CO*⋯O5*B* ^vi^	0.80 (4)	2.03 (4)	2.823 (3)	173 (3)
O4*C*—H4*CO*⋯O5*C* ^i^	0.80 (4)	1.97 (4)	2.738 (3)	162 (4)
N3*D*—H6*ND*⋯N4*C* ^vii^	0.92 (4)	2.62 (4)	3.268 (4)	128 (3)
N3*D*—H6*ND*⋯O5*D*	0.92 (4)	2.36 (4)	2.972 (4)	124 (3)
N4*D*—H4*ND*⋯N2*B* ^i^	0.89 (4)	2.26 (4)	3.075 (4)	153 (3)
N4*D*—H5*ND*⋯O3*D* ^i^	0.87 (4)	2.09 (4)	2.957 (3)	176 (3)
O3*D*—H3*DO*⋯O4*B* ^viii^	0.90 (4)	1.82 (4)	2.711 (3)	171 (3)
O4*D*—H4*DO*⋯O5*D* ^iii^	0.79 (3)	2.06 (3)	2.822 (3)	164 (4)

Symmetry codes: (i) 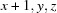; (ii) 
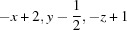
; (iii) 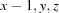; (iv) 
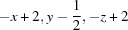
; (v) 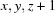; (vi) 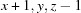; (vii) 
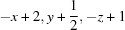
; (viii) 
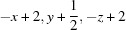
.

**Table 2:** Parameters (Å^2^, Å^3^) of the Hirshfeld surface of the four crystallographically independent mol­ecules *A*, *B*, *C* and *D*)

Mol­ecule	Volume	Area	Globularity	Asphericity
*A*	356.33	322.48	0.754	0.144
*B*	348.71	318.70	0.752	0.142
*C*	349.46	317.26	0.756	0.144
*D*	355.80	323.52	0.751	0.141

Hirshfeld surface analysis was performed using the program *CrystalExplorer* (Wolff *et al.* 2012 ▸).

**Table 3:** Experimental details

Crystal data	
Chemical formula	C_12_H_20_N_4_O_5_
*M*_r_	300.32
Crystal system, space group	Monoclinic, *P*2_1_
Temperature (K)	293
*a*, *b*, *c* (Å)	11.627 (4), 18.929 (4), 13.085 (3)
β (°)	93.67 (2)
*V* (Å^3^)	2873.9 (13)
*Z*	8
Radiation type	Mo *K*α
μ (mm^−1^)	0.11
Crystal size (mm)	0.40 × 0.25 × 0.25

Data collection	
Diffractometer	Bruker–Nonius KappaCCD
Absorption correction	Multi-scan (*SADABS*; Bruker, 2001 ▸)
*T*_min_, *T*_max_	0.945, 0.961
No. of measured, independent and observed [*I* > 2σ(*I*)] reflections	21683, 11330, 9391
*R*_int_	0.030
(sin θ/λ)_max_ (Å^−1^)	0.650

Refinement	
*R*[*F* ^2^ > 2σ(*F* ^2^)], *wR*(*F* ^2^), *S*	0.039, 0.087, 1.06
No. of reflections	11330
No. of parameters	838
No. of restraints	1
H-atom treatment	H atoms treated by a mixture of independent and constrained refinement
Δρ_max_, Δρ_min_ (e Å^−3^)	0.21, −0.24
Absolute structure	Flack *x* determined using 3518 quotients [(*I* ^+^)−(*I* ^−^)]/[(*I* ^+^)+(*I* ^−^)] (Parsons *et al.*, 2013 ▸)
Absolute structure parameter	0.1 (3)

Computer programs: *COLLECT* (Nonius, 1999 ▸), *DIRAX/LSQ* (Duisenberg *et al.*, 2000 ▸), *EVALCCD* (Duisenberg *et al.*, 2003 ▸), *SIR97* (Altomare *et al.*, 1999 ▸), *SHELXL2016* (Sheldrick, 2015 ▸), *ORTEP-3 for Windows* and *WinGX* (Farrugia, 2012 ▸) and *Mercury* (Macrae *et al.*, 2008 ▸).

## supplementary crystallographic information

### Crystal data

**Table:**

C_12_H_20_N_4_O_5_	*F*(000) = 1280
*M**_r_* = 300.32	*D*_x_ = 1.388 Mg m^−^^3^
Monoclinic, *P*2_1_	Mo *K*α radiation, λ = 0.71073 Å
*a* = 11.627 (4) Å	Cell parameters from 93 reflections
*b* = 18.929 (4) Å	θ = 2.7–23.4°
*c* = 13.085 (3) Å	µ = 0.11 mm^−^^1^
β = 93.67 (2)°	*T* = 293 K
*V* = 2873.9 (13) Å^3^	Prism, pale yellow
*Z* = 8	0.40 × 0.25 × 0.25 mm

### Data collection

**Table:**

Bruker–Nonius KappaCCD diffractometer	11330 independent reflections
Radiation source: normal-focus sealed tube	9391 reflections with *I* > 2σ(*I*)
Graphite monochromator	*R*_int_ = 0.030
Detector resolution: 9 pixels mm^-1^	θ_max_ = 27.5°, θ_min_ = 3.1°
CCD rotation images, thick slices scans	*h* = −14→15
Absorption correction: multi-scan (*SADABS*; Bruker, 2001)	*k* = −24→22
*T*_min_ = 0.945, *T*_max_ = 0.961	*l* = −15→16
21683 measured reflections	

### Refinement

**Table:**

Refinement on *F*^2^	Secondary atom site location: difference Fourier map
Least-squares matrix: full	Hydrogen site location: mixed
*R*[*F*^2^ > 2σ(*F*^2^)] = 0.039	H atoms treated by a mixture of independent and constrained refinement
*wR*(*F*^2^) = 0.087	*w* = 1/[σ^2^(*F*_o_^2^) + (0.0372*P*)^2^ + 0.3518*P*] where *P* = (*F*_o_^2^ + 2*F*_c_^2^)/3
*S* = 1.06	(Δ/σ)_max_ < 0.001
11330 reflections	Δρ_max_ = 0.21 e Å^−^^3^
838 parameters	Δρ_min_ = −0.24 e Å^−^^3^
1 restraint	Absolute structure: Flack *x* determined using 3518 quotients [(*I*^+^)-(*I*^-^)]/[(*I*^+^)+(*I*^-^)] (Parsons *et al.*, 2013)
Primary atom site location: structure-invariant direct methods	Absolute structure parameter: 0.1 (3)

### Special details

**Table:**

Geometry. All esds (except the esd in the dihedral angle between two l.s. planes) are estimated using the full covariance matrix. The cell esds are taken into account individually in the estimation of esds in distances, angles and torsion angles; correlations between esds in cell parameters are only used when they are defined by crystal symmetry. An approximate (isotropic) treatment of cell esds is used for estimating esds involving l.s. planes.

### Fractional atomic coordinates and isotropic or equivalent isotropic displacement parameters (Å^2^)

**Table:**

	*x*	*y*	*z*	*U*_iso_*/*U*_eq_	
C1A	0.4499 (2)	0.10494 (19)	0.8668 (2)	0.0385 (7)	
C2A	0.4978 (3)	0.1789 (2)	0.8642 (3)	0.0571 (10)	
H2A1	0.575335	0.179182	0.894136	0.086*	
H2A2	0.451041	0.209892	0.902301	0.086*	
H2A3	0.497375	0.194853	0.794516	0.086*	
C3A	0.4644 (4)	0.0711 (3)	0.9697 (3)	0.0666 (12)	
H3A1	0.432081	0.024388	0.966423	0.100*	
H3A2	0.425627	0.098770	1.018427	0.100*	
H3A3	0.544978	0.068187	0.990639	0.100*	
C4A	0.4311 (2)	0.06262 (17)	0.7036 (2)	0.0293 (6)	
H4A	0.445038	0.106956	0.667761	0.035*	
C5A	0.3109 (2)	0.06458 (16)	0.7440 (2)	0.0298 (6)	
H5A	0.258848	0.089654	0.694582	0.036*	
C6A	0.2590 (2)	−0.00699 (17)	0.7667 (2)	0.0302 (6)	
H6A	0.319112	−0.038252	0.797012	0.036*	
C7A	0.1629 (2)	0.00012 (18)	0.8393 (2)	0.0387 (7)	
H7A1	0.194368	0.019380	0.903860	0.046*	
H7A2	0.105899	0.033201	0.810526	0.046*	
C8A	0.4623 (2)	0.00136 (18)	0.6370 (2)	0.0331 (7)	
H8A1	0.445951	−0.042790	0.670792	0.040*	
H8A2	0.416391	0.003080	0.572510	0.040*	
C9A	0.6330 (2)	0.04647 (18)	0.5475 (2)	0.0346 (7)	
H9A	0.590944	0.075309	0.501233	0.042*	
C10A	0.6729 (2)	−0.02857 (17)	0.6727 (2)	0.0304 (6)	
C11A	0.7716 (2)	−0.00571 (16)	0.6308 (2)	0.0296 (6)	
C12A	0.8881 (2)	−0.02684 (17)	0.6631 (2)	0.0311 (7)	
N1A	0.58465 (18)	0.00469 (14)	0.61784 (18)	0.0316 (6)	
N2A	0.74518 (18)	0.04130 (14)	0.55249 (19)	0.0338 (6)	
N3A	0.6553 (3)	−0.07792 (17)	0.7459 (2)	0.0467 (7)	
H3NA	0.721 (3)	−0.091 (2)	0.780 (3)	0.056*	
H6NA	0.603 (3)	−0.066 (2)	0.790 (3)	0.056*	
N4A	0.9726 (2)	0.00707 (17)	0.6193 (2)	0.0397 (7)	
H4NA	1.047 (3)	−0.0066 (19)	0.636 (3)	0.048*	
H5NA	0.958 (3)	0.038 (2)	0.567 (3)	0.048*	
O1A	0.50240 (15)	0.06020 (12)	0.79547 (15)	0.0346 (5)	
O2A	0.32999 (16)	0.10699 (12)	0.83281 (16)	0.0398 (5)	
O3A	0.21584 (17)	−0.03486 (13)	0.67078 (17)	0.0368 (5)	
H3AO	0.241 (3)	−0.073 (2)	0.663 (3)	0.044*	
O4A	0.10827 (18)	−0.06497 (14)	0.8579 (2)	0.0475 (6)	
H4AO	0.058 (3)	−0.069 (2)	0.813 (3)	0.057*	
O5A	0.90664 (16)	−0.07293 (12)	0.72975 (18)	0.0415 (5)	
C1B	0.8545 (2)	0.12784 (18)	0.8964 (2)	0.0347 (7)	
C2B	0.8112 (3)	0.1470 (2)	0.7896 (3)	0.0528 (9)	
H2B1	0.732425	0.132345	0.778265	0.079*	
H2B2	0.857329	0.123823	0.741388	0.079*	
H2B3	0.816254	0.197275	0.780724	0.079*	
C3B	0.8344 (4)	0.0528 (2)	0.9203 (3)	0.0719 (13)	
H3B1	0.863932	0.042953	0.989045	0.108*	
H3B2	0.873014	0.023520	0.873378	0.108*	
H3B3	0.753202	0.043148	0.914104	0.108*	
C4B	0.9924 (2)	0.20415 (18)	0.9727 (2)	0.0321 (7)	
H4B	1.036373	0.238884	0.935659	0.039*	
C5B	0.8700 (2)	0.23281 (16)	0.9824 (2)	0.0289 (6)	
H5B	0.850109	0.264722	0.924945	0.035*	
C6B	1.0590 (2)	0.18687 (16)	1.0732 (2)	0.0295 (6)	
H6B	1.006888	0.165343	1.120276	0.035*	
C7B	1.1590 (2)	0.13783 (18)	1.0591 (2)	0.0368 (7)	
H7B1	1.130751	0.094330	1.027369	0.044*	
H7B2	1.211537	0.159674	1.013941	0.044*	
C8B	0.8436 (2)	0.26854 (17)	1.0808 (2)	0.0307 (6)	
H8B1	0.866228	0.237940	1.138031	0.037*	
H8B2	0.887856	0.311879	1.088399	0.037*	
C9B	0.6687 (2)	0.34498 (17)	1.0474 (2)	0.0362 (7)	
H9B	0.707578	0.382321	1.018851	0.043*	
C10B	0.6377 (2)	0.24188 (16)	1.1177 (2)	0.0267 (6)	
C11B	0.5373 (2)	0.28025 (16)	1.1038 (2)	0.0282 (6)	
C12B	0.4237 (2)	0.25680 (17)	1.1263 (2)	0.0287 (6)	
N1B	0.72083 (17)	0.28476 (13)	1.08206 (18)	0.0292 (5)	
N2B	0.55807 (19)	0.34474 (14)	1.0585 (2)	0.0363 (6)	
N3B	0.6610 (2)	0.17694 (15)	1.1577 (2)	0.0364 (6)	
H3NB	0.713 (3)	0.152 (2)	1.125 (3)	0.044*	
H6NB	0.596 (3)	0.148 (2)	1.168 (2)	0.044*	
N4B	0.3352 (2)	0.29355 (16)	1.0870 (2)	0.0374 (7)	
H4NB	0.266 (3)	0.2772 (19)	1.100 (2)	0.045*	
H5NB	0.343 (3)	0.331 (2)	1.052 (3)	0.045*	
O1B	0.80252 (15)	0.17051 (11)	0.97070 (15)	0.0335 (5)	
O2B	0.97407 (18)	0.14387 (15)	0.9101 (2)	0.0589 (8)	
O3B	1.09799 (15)	0.25348 (11)	1.11297 (17)	0.0314 (5)	
H3BO	1.105 (3)	0.2541 (19)	1.180 (3)	0.038*	
O4B	1.21881 (17)	0.12216 (12)	1.15462 (17)	0.0399 (5)	
H4BO	1.279 (3)	0.153 (2)	1.160 (3)	0.048*	
O5B	0.41145 (15)	0.20287 (12)	1.18070 (16)	0.0358 (5)	
C1C	1.3293 (2)	0.31702 (17)	0.6014 (2)	0.0326 (7)	
C2C	1.2971 (3)	0.2956 (2)	0.7064 (3)	0.0478 (9)	
H2C1	1.220794	0.312200	0.717152	0.072*	
H2C2	1.350733	0.315989	0.756897	0.072*	
H2C3	1.299256	0.245100	0.712097	0.072*	
C3C	1.3097 (4)	0.3939 (2)	0.5819 (3)	0.0616 (10)	
H3C1	1.330361	0.405345	0.513994	0.092*	
H3C2	1.356290	0.420972	0.630854	0.092*	
H3C3	1.229884	0.404856	0.588373	0.092*	
C4C	1.4572 (2)	0.24411 (17)	0.5198 (2)	0.0306 (6)	
H4C	1.507203	0.207539	0.551738	0.037*	
C5C	1.3333 (2)	0.21551 (16)	0.5076 (2)	0.0274 (6)	
H5C	1.321698	0.182162	0.563334	0.033*	
C6C	1.5083 (2)	0.26831 (16)	0.4210 (2)	0.0281 (6)	
H6C	1.450099	0.293888	0.378066	0.034*	
C7C	1.6126 (2)	0.31482 (18)	0.4441 (2)	0.0363 (7)	
H7C1	1.589874	0.356104	0.481789	0.044*	
H7C2	1.669328	0.289062	0.486974	0.044*	
C8C	1.2928 (2)	0.18192 (17)	0.4079 (2)	0.0312 (7)	
H8C1	1.304723	0.214266	0.352052	0.037*	
H8C2	1.337192	0.139478	0.397219	0.037*	
C9C	1.1244 (2)	0.10359 (17)	0.4449 (2)	0.0341 (7)	
H9C	1.168211	0.066232	0.472304	0.041*	
C10C	1.0799 (2)	0.20609 (16)	0.3757 (2)	0.0265 (6)	
C11C	0.9828 (2)	0.16832 (16)	0.3938 (2)	0.0275 (6)	
C12C	0.8653 (2)	0.19404 (17)	0.3728 (2)	0.0284 (6)	
N1C	1.17044 (17)	0.16400 (13)	0.40830 (18)	0.0287 (5)	
N2C	1.01193 (18)	0.10398 (14)	0.4372 (2)	0.0349 (6)	
N3C	1.0940 (2)	0.27011 (15)	0.3283 (2)	0.0343 (6)	
H3NC	1.147 (3)	0.295 (2)	0.361 (3)	0.041*	
H6NC	1.026 (3)	0.2926 (19)	0.320 (3)	0.041*	
N4C	0.7807 (2)	0.15465 (17)	0.4048 (2)	0.0371 (6)	
H4NC	0.794 (3)	0.119 (2)	0.445 (3)	0.044*	
H5NC	0.712 (3)	0.1667 (19)	0.394 (3)	0.044*	
O1C	1.26738 (15)	0.27748 (11)	0.52323 (14)	0.0321 (5)	
O2C	1.44747 (17)	0.30005 (15)	0.59017 (18)	0.0517 (7)	
O3C	1.54041 (15)	0.20510 (12)	0.37092 (16)	0.0314 (5)	
H3CO	1.508 (3)	0.202 (2)	0.315 (3)	0.038*	
O4C	1.66330 (18)	0.33696 (13)	0.35309 (18)	0.0408 (6)	
H4CO	1.706 (3)	0.305 (2)	0.343 (3)	0.049*	
O5C	0.84870 (16)	0.25104 (12)	0.32754 (16)	0.0370 (5)	
C1D	0.9304 (2)	0.34582 (18)	0.6288 (2)	0.0365 (7)	
C2D	0.9758 (3)	0.2712 (2)	0.6311 (3)	0.0514 (9)	
H2D1	1.049044	0.269986	0.601224	0.077*	
H2D2	0.922455	0.241058	0.592750	0.077*	
H2D3	0.984696	0.255065	0.700677	0.077*	
C3D	0.9309 (3)	0.3795 (3)	0.5249 (3)	0.0594 (10)	
H3D1	0.903472	0.427154	0.528558	0.089*	
H3D2	0.881677	0.353155	0.477111	0.089*	
H3D3	1.008047	0.379545	0.502704	0.089*	
C4D	0.8097 (2)	0.38604 (16)	0.7520 (2)	0.0298 (6)	
H4D	0.764469	0.360525	0.800842	0.036*	
C5D	0.9361 (2)	0.38703 (17)	0.7923 (2)	0.0285 (6)	
H5D	0.954839	0.342267	0.827122	0.034*	
C6D	0.7563 (2)	0.45837 (16)	0.7322 (2)	0.0285 (6)	
H6D	0.813951	0.490026	0.705506	0.034*	
C7D	0.6519 (2)	0.45495 (18)	0.6564 (2)	0.0358 (7)	
H7D1	0.675380	0.437577	0.591227	0.043*	
H7D2	0.596325	0.421923	0.681327	0.043*	
C8D	0.9789 (2)	0.44773 (17)	0.8599 (2)	0.0319 (7)	
H8D1	0.957747	0.492273	0.827178	0.038*	
H8D2	0.943146	0.445603	0.924822	0.038*	
C9D	1.1624 (2)	0.40135 (18)	0.9468 (2)	0.0345 (7)	
H9D	1.127080	0.371585	0.991886	0.041*	
C10D	1.1837 (2)	0.47887 (16)	0.8249 (2)	0.0287 (6)	
C11D	1.2887 (2)	0.45647 (16)	0.8665 (2)	0.0292 (6)	
C12D	1.4012 (2)	0.47614 (16)	0.8326 (2)	0.0300 (6)	
N1D	1.10368 (18)	0.44364 (14)	0.87749 (18)	0.0296 (5)	
N2D	1.27366 (18)	0.40725 (14)	0.94288 (19)	0.0337 (6)	
N3D	1.1549 (2)	0.52948 (17)	0.7538 (2)	0.0434 (7)	
H3ND	1.101 (3)	0.517 (2)	0.710 (3)	0.052*	
H6ND	1.215 (3)	0.546 (2)	0.719 (3)	0.052*	
N4D	1.4901 (2)	0.43900 (16)	0.8729 (2)	0.0383 (7)	
H4ND	1.484 (3)	0.410 (2)	0.925 (3)	0.046*	
H5ND	1.559 (3)	0.454 (2)	0.862 (3)	0.046*	
O1D	0.99370 (15)	0.39015 (12)	0.69983 (15)	0.0341 (5)	
O2D	0.81508 (16)	0.34503 (12)	0.66228 (17)	0.0407 (5)	
O3D	0.72582 (16)	0.48331 (12)	0.82927 (15)	0.0323 (5)	
H3DO	0.739 (3)	0.530 (2)	0.829 (3)	0.039*	
O4D	0.59912 (19)	0.52187 (13)	0.64195 (19)	0.0426 (6)	
H4DO	0.554 (3)	0.528 (2)	0.684 (3)	0.040 (10)*	
O5D	1.41082 (16)	0.52327 (12)	0.76776 (16)	0.0398 (5)	

### Atomic displacement parameters (Å^2^)

**Table:**

	*U*^11^	*U*^22^	*U*^33^	*U*^12^	*U*^13^	*U*^23^
C1A	0.0323 (15)	0.047 (2)	0.0357 (18)	0.0033 (14)	−0.0020 (12)	−0.0069 (15)
C2A	0.0444 (19)	0.052 (3)	0.074 (3)	−0.0073 (17)	−0.0035 (17)	−0.023 (2)
C3A	0.069 (3)	0.093 (4)	0.037 (2)	0.013 (2)	−0.0004 (16)	0.002 (2)
C4A	0.0218 (12)	0.0339 (18)	0.0321 (16)	0.0003 (11)	0.0008 (10)	0.0044 (13)
C5A	0.0239 (13)	0.0330 (18)	0.0323 (16)	0.0037 (12)	0.0004 (10)	0.0002 (13)
C6A	0.0229 (12)	0.0310 (18)	0.0365 (16)	0.0040 (11)	0.0005 (10)	−0.0031 (13)
C7A	0.0320 (15)	0.042 (2)	0.0428 (18)	−0.0028 (14)	0.0083 (12)	−0.0010 (15)
C8A	0.0208 (12)	0.043 (2)	0.0364 (16)	−0.0021 (12)	0.0051 (11)	−0.0032 (14)
C9A	0.0265 (14)	0.039 (2)	0.0384 (17)	0.0016 (12)	0.0024 (11)	0.0047 (14)
C10A	0.0262 (13)	0.0339 (18)	0.0310 (16)	0.0006 (12)	0.0017 (11)	−0.0015 (13)
C11A	0.0263 (13)	0.0273 (17)	0.0352 (16)	−0.0017 (11)	0.0028 (11)	−0.0005 (13)
C12A	0.0284 (14)	0.0283 (18)	0.0363 (17)	0.0019 (12)	0.0006 (11)	−0.0046 (14)
N1A	0.0228 (11)	0.0378 (16)	0.0346 (13)	−0.0016 (10)	0.0039 (9)	0.0014 (11)
N2A	0.0257 (11)	0.0371 (16)	0.0388 (14)	−0.0014 (10)	0.0029 (9)	0.0035 (12)
N3A	0.0396 (15)	0.052 (2)	0.0491 (18)	−0.0022 (13)	0.0041 (13)	0.0154 (15)
N4A	0.0233 (12)	0.0489 (19)	0.0470 (17)	0.0030 (12)	0.0038 (11)	0.0074 (14)
O1A	0.0268 (10)	0.0421 (14)	0.0340 (11)	0.0047 (8)	−0.0053 (8)	−0.0050 (10)
O2A	0.0305 (10)	0.0437 (15)	0.0454 (13)	0.0002 (9)	0.0034 (8)	−0.0168 (11)
O3A	0.0259 (10)	0.0373 (14)	0.0467 (13)	0.0042 (9)	−0.0013 (8)	−0.0116 (11)
O4A	0.0326 (11)	0.0525 (17)	0.0572 (16)	−0.0042 (11)	0.0017 (10)	0.0204 (13)
O5A	0.0321 (11)	0.0388 (14)	0.0532 (14)	0.0023 (9)	−0.0007 (9)	0.0085 (11)
C1B	0.0330 (15)	0.040 (2)	0.0317 (17)	−0.0031 (13)	0.0040 (11)	−0.0041 (14)
C2B	0.061 (2)	0.060 (3)	0.036 (2)	−0.0034 (19)	−0.0016 (15)	−0.0063 (18)
C3B	0.119 (4)	0.042 (3)	0.058 (3)	−0.005 (2)	0.030 (2)	−0.006 (2)
C4B	0.0233 (12)	0.0389 (19)	0.0345 (17)	0.0001 (12)	0.0050 (11)	−0.0013 (14)
C5B	0.0222 (13)	0.0328 (18)	0.0314 (16)	−0.0008 (11)	0.0002 (10)	0.0016 (12)
C6B	0.0225 (12)	0.0285 (17)	0.0377 (16)	−0.0012 (11)	0.0040 (11)	−0.0006 (13)
C7B	0.0277 (14)	0.0365 (19)	0.0460 (19)	0.0046 (12)	0.0012 (12)	−0.0029 (15)
C8B	0.0179 (12)	0.0331 (18)	0.0407 (16)	0.0017 (11)	−0.0008 (10)	−0.0063 (14)
C9B	0.0256 (14)	0.0318 (18)	0.0510 (19)	−0.0031 (12)	0.0020 (12)	0.0024 (15)
C10B	0.0238 (12)	0.0279 (17)	0.0281 (15)	−0.0013 (11)	0.0003 (10)	−0.0038 (12)
C11B	0.0235 (13)	0.0297 (17)	0.0315 (15)	−0.0017 (11)	0.0011 (10)	−0.0003 (13)
C12B	0.0238 (12)	0.0340 (18)	0.0281 (15)	−0.0022 (12)	−0.0002 (10)	−0.0043 (13)
N1B	0.0207 (10)	0.0285 (15)	0.0384 (14)	0.0009 (9)	0.0009 (9)	−0.0037 (11)
N2B	0.0234 (11)	0.0359 (16)	0.0496 (16)	0.0017 (10)	0.0006 (10)	0.0057 (13)
N3B	0.0307 (13)	0.0323 (17)	0.0463 (16)	0.0027 (11)	0.0038 (11)	0.0019 (13)
N4B	0.0212 (12)	0.0409 (18)	0.0499 (17)	−0.0006 (11)	0.0020 (10)	0.0123 (13)
O1B	0.0311 (10)	0.0354 (13)	0.0348 (11)	−0.0087 (9)	0.0073 (8)	−0.0068 (9)
O2B	0.0325 (12)	0.0735 (19)	0.0694 (17)	0.0105 (11)	−0.0068 (10)	−0.0422 (15)
O3B	0.0263 (9)	0.0347 (13)	0.0331 (11)	−0.0006 (8)	0.0018 (8)	−0.0042 (10)
O4B	0.0298 (10)	0.0345 (14)	0.0546 (14)	0.0009 (9)	−0.0040 (9)	0.0089 (11)
O5B	0.0276 (9)	0.0398 (14)	0.0397 (12)	−0.0025 (9)	0.0001 (8)	0.0077 (10)
C1C	0.0314 (14)	0.0380 (19)	0.0286 (16)	0.0002 (13)	0.0033 (11)	−0.0046 (14)
C2C	0.0516 (19)	0.054 (2)	0.0383 (19)	−0.0004 (16)	0.0070 (14)	−0.0019 (17)
C3C	0.098 (3)	0.040 (2)	0.046 (2)	−0.004 (2)	−0.0083 (19)	−0.0042 (19)
C4C	0.0227 (12)	0.0349 (18)	0.0340 (16)	0.0005 (12)	−0.0004 (10)	−0.0057 (13)
C5C	0.0214 (12)	0.0280 (16)	0.0332 (16)	0.0007 (11)	0.0045 (10)	0.0027 (12)
C6C	0.0218 (12)	0.0279 (16)	0.0346 (15)	0.0022 (11)	0.0010 (10)	−0.0027 (13)
C7C	0.0323 (15)	0.0350 (19)	0.0416 (18)	−0.0077 (13)	0.0036 (12)	−0.0020 (15)
C8C	0.0195 (12)	0.0332 (18)	0.0417 (17)	−0.0034 (11)	0.0077 (11)	−0.0074 (14)
C9C	0.0264 (13)	0.0317 (18)	0.0441 (18)	−0.0010 (12)	0.0020 (11)	0.0023 (14)
C10C	0.0264 (12)	0.0259 (16)	0.0275 (15)	−0.0012 (11)	0.0035 (10)	−0.0019 (12)
C11C	0.0227 (12)	0.0297 (17)	0.0300 (15)	−0.0008 (11)	0.0012 (10)	−0.0018 (12)
C12C	0.0252 (13)	0.0317 (18)	0.0285 (15)	0.0003 (11)	0.0024 (10)	−0.0026 (13)
N1C	0.0206 (10)	0.0290 (15)	0.0369 (14)	−0.0025 (9)	0.0041 (9)	−0.0045 (11)
N2C	0.0247 (11)	0.0326 (16)	0.0476 (16)	−0.0020 (10)	0.0025 (10)	0.0033 (12)
N3C	0.0318 (12)	0.0293 (15)	0.0422 (15)	−0.0037 (11)	0.0068 (10)	0.0006 (12)
N4C	0.0220 (11)	0.0400 (18)	0.0494 (17)	0.0021 (11)	0.0030 (11)	0.0129 (13)
O1C	0.0252 (9)	0.0371 (13)	0.0338 (11)	0.0045 (8)	0.0002 (7)	−0.0097 (10)
O2C	0.0311 (11)	0.0708 (19)	0.0538 (15)	−0.0093 (11)	0.0077 (9)	−0.0328 (13)
O3C	0.0235 (9)	0.0374 (13)	0.0330 (11)	0.0025 (8)	−0.0010 (8)	−0.0078 (10)
O4C	0.0316 (11)	0.0348 (14)	0.0571 (14)	−0.0018 (9)	0.0117 (9)	0.0072 (11)
O5C	0.0297 (10)	0.0379 (14)	0.0435 (12)	0.0012 (9)	0.0038 (8)	0.0063 (11)
C1D	0.0310 (15)	0.042 (2)	0.0371 (17)	−0.0042 (13)	0.0047 (12)	−0.0068 (15)
C2D	0.0472 (19)	0.045 (2)	0.062 (2)	0.0059 (16)	0.0096 (16)	−0.0112 (19)
C3D	0.070 (2)	0.067 (3)	0.041 (2)	−0.008 (2)	0.0042 (16)	0.003 (2)
C4D	0.0234 (12)	0.0316 (18)	0.0345 (16)	−0.0045 (12)	0.0038 (10)	−0.0010 (13)
C5D	0.0237 (12)	0.0291 (17)	0.0328 (16)	0.0016 (11)	0.0037 (10)	0.0037 (13)
C6D	0.0208 (12)	0.0315 (17)	0.0331 (15)	−0.0013 (11)	0.0016 (10)	0.0018 (13)
C7D	0.0294 (14)	0.041 (2)	0.0365 (17)	0.0016 (13)	−0.0039 (11)	0.0001 (14)
C8D	0.0204 (12)	0.0398 (19)	0.0351 (16)	0.0019 (12)	−0.0002 (10)	−0.0013 (14)
C9D	0.0272 (14)	0.040 (2)	0.0359 (17)	−0.0022 (12)	−0.0004 (11)	0.0079 (14)
C10D	0.0271 (13)	0.0289 (17)	0.0296 (15)	0.0001 (11)	−0.0011 (10)	−0.0008 (13)
C11D	0.0253 (13)	0.0289 (18)	0.0329 (16)	0.0000 (11)	−0.0004 (11)	−0.0010 (13)
C12D	0.0254 (13)	0.0298 (18)	0.0347 (17)	−0.0018 (11)	0.0008 (11)	−0.0068 (14)
N1D	0.0230 (11)	0.0350 (15)	0.0303 (13)	−0.0014 (10)	−0.0017 (9)	0.0013 (11)
N2D	0.0250 (11)	0.0363 (16)	0.0393 (15)	0.0002 (10)	−0.0009 (9)	0.0036 (12)
N3D	0.0352 (14)	0.050 (2)	0.0444 (17)	0.0022 (13)	−0.0009 (11)	0.0160 (15)
N4D	0.0226 (12)	0.0458 (19)	0.0462 (17)	−0.0010 (12)	0.0007 (11)	0.0083 (14)
O1D	0.0270 (9)	0.0415 (14)	0.0342 (11)	−0.0057 (9)	0.0061 (8)	−0.0043 (10)
O2D	0.0257 (10)	0.0454 (15)	0.0508 (14)	−0.0007 (9)	0.0009 (8)	−0.0194 (11)
O3D	0.0284 (10)	0.0327 (13)	0.0359 (12)	−0.0017 (9)	0.0023 (8)	−0.0048 (10)
O4D	0.0323 (11)	0.0474 (16)	0.0482 (14)	0.0074 (10)	0.0033 (10)	0.0143 (12)
O5D	0.0310 (10)	0.0410 (15)	0.0477 (13)	−0.0018 (9)	0.0056 (9)	0.0085 (11)

### Geometric parameters (Å, º)

**Table:**

C1A—O1A	1.426 (4)	C1C—O1C	1.425 (4)
C1A—O2A	1.437 (3)	C1C—O2C	1.428 (3)
C1A—C3A	1.492 (5)	C1C—C3C	1.492 (5)
C1A—C2A	1.508 (5)	C1C—C2C	1.502 (4)
C2A—H2A1	0.9600	C2C—H2C1	0.9600
C2A—H2A2	0.9600	C2C—H2C2	0.9600
C2A—H2A3	0.9600	C2C—H2C3	0.9600
C3A—H3A1	0.9600	C3C—H3C1	0.9600
C3A—H3A2	0.9600	C3C—H3C2	0.9600
C3A—H3A3	0.9600	C3C—H3C3	0.9600
C4A—O1A	1.417 (3)	C4C—O2C	1.413 (4)
C4A—C8A	1.509 (4)	C4C—C6C	1.526 (4)
C4A—C5A	1.525 (4)	C4C—C5C	1.538 (4)
C4A—H4A	0.9800	C4C—H4C	0.9800
C5A—O2A	1.418 (4)	C5C—O1C	1.423 (3)
C5A—C6A	1.520 (4)	C5C—C8C	1.501 (4)
C5A—H5A	0.9800	C5C—H5C	0.9800
C6A—O3A	1.423 (4)	C6C—O3C	1.426 (3)
C6A—C7A	1.518 (4)	C6C—C7C	1.513 (4)
C6A—H6A	0.9800	C6C—H6C	0.9800
C7A—O4A	1.414 (4)	C7C—O4C	1.425 (4)
C7A—H7A1	0.9700	C7C—H7C1	0.9700
C7A—H7A2	0.9700	C7C—H7C2	0.9700
C8A—N1A	1.461 (3)	C8C—N1C	1.463 (3)
C8A—H8A1	0.9700	C8C—H8C1	0.9700
C8A—H8A2	0.9700	C8C—H8C2	0.9700
C9A—N2A	1.306 (3)	C9C—N2C	1.305 (3)
C9A—N1A	1.362 (4)	C9C—N1C	1.363 (4)
C9A—H9A	0.9300	C9C—H9C	0.9300
C10A—N3A	1.363 (4)	C10C—N1C	1.366 (4)
C10A—N1A	1.367 (4)	C10C—C11C	1.370 (4)
C10A—C11A	1.373 (4)	C10C—N3C	1.376 (4)
C11A—N2A	1.377 (4)	C11C—N2C	1.377 (4)
C11A—C12A	1.449 (4)	C11C—C12C	1.460 (4)
C12A—O5A	1.243 (4)	C12C—O5C	1.240 (4)
C12A—N4A	1.334 (4)	C12C—N4C	1.323 (4)
N3A—H3NA	0.89 (4)	N3C—H3NC	0.87 (4)
N3A—H6NA	0.89 (4)	N3C—H6NC	0.90 (4)
N4A—H4NA	0.92 (4)	N4C—H4NC	0.86 (4)
N4A—H5NA	0.91 (4)	N4C—H5NC	0.84 (4)
O3A—H3AO	0.79 (4)	O3C—H3CO	0.80 (4)
O4A—H4AO	0.80 (4)	O4C—H4CO	0.80 (4)
C1B—O2B	1.423 (4)	C1D—O1D	1.422 (4)
C1B—O1B	1.427 (3)	C1D—O2D	1.437 (3)
C1B—C3B	1.476 (5)	C1D—C3D	1.501 (5)
C1B—C2B	1.500 (5)	C1D—C2D	1.507 (5)
C2B—H2B1	0.9600	C2D—H2D1	0.9600
C2B—H2B2	0.9600	C2D—H2D2	0.9600
C2B—H2B3	0.9600	C2D—H2D3	0.9600
C3B—H3B1	0.9600	C3D—H3D1	0.9600
C3B—H3B2	0.9600	C3D—H3D2	0.9600
C3B—H3B3	0.9600	C3D—H3D3	0.9600
C4B—O2B	1.413 (4)	C4D—O2D	1.412 (3)
C4B—C6B	1.518 (4)	C4D—C6D	1.519 (4)
C4B—C5B	1.536 (4)	C4D—C5D	1.530 (4)
C4B—H4B	0.9800	C4D—H4D	0.9800
C5B—O1B	1.419 (3)	C5D—O1D	1.421 (3)
C5B—C8B	1.503 (4)	C5D—C8D	1.515 (4)
C5B—H5B	0.9800	C5D—H5D	0.9800
C6B—O3B	1.427 (4)	C6D—O3D	1.421 (3)
C6B—C7B	1.508 (4)	C6D—C7D	1.519 (4)
C6B—H6B	0.9800	C6D—H6D	0.9800
C7B—O4B	1.422 (4)	C7D—O4D	1.415 (4)
C7B—H7B1	0.9700	C7D—H7D1	0.9700
C7B—H7B2	0.9700	C7D—H7D2	0.9700
C8B—N1B	1.462 (3)	C8D—N1D	1.457 (3)
C8B—H8B1	0.9700	C8D—H8D1	0.9700
C8B—H8B2	0.9700	C8D—H8D2	0.9700
C9B—N2B	1.304 (4)	C9D—N2D	1.303 (3)
C9B—N1B	1.355 (4)	C9D—N1D	1.360 (4)
C9B—H9B	0.9300	C9D—H9D	0.9300
C10B—N3B	1.357 (4)	C10D—N3D	1.362 (4)
C10B—N1B	1.367 (4)	C10D—N1D	1.366 (4)
C10B—C11B	1.377 (4)	C10D—C11D	1.372 (4)
C11B—N2B	1.385 (4)	C11D—N2D	1.385 (4)
C11B—C12B	1.441 (4)	C11D—C12D	1.456 (4)
C12B—O5B	1.258 (3)	C12D—O5D	1.241 (4)
C12B—N4B	1.319 (4)	C12D—N4D	1.332 (4)
N3B—H3NB	0.89 (3)	N3D—H3ND	0.86 (4)
N3B—H6NB	0.95 (4)	N3D—H6ND	0.92 (4)
N4B—H4NB	0.89 (4)	N4D—H4ND	0.89 (4)
N4B—H5NB	0.85 (4)	N4D—H5ND	0.87 (4)
O3B—H3BO	0.87 (3)	O3D—H3DO	0.90 (4)
O4B—H4BO	0.91 (4)	O4D—H4DO	0.79 (3)
			
O1A—C1A—O2A	105.1 (2)	O1C—C1C—O2C	104.7 (2)
O1A—C1A—C3A	107.9 (3)	O1C—C1C—C3C	109.0 (3)
O2A—C1A—C3A	110.0 (3)	O2C—C1C—C3C	109.8 (3)
O1A—C1A—C2A	111.3 (3)	O1C—C1C—C2C	111.8 (3)
O2A—C1A—C2A	108.7 (3)	O2C—C1C—C2C	109.3 (3)
C3A—C1A—C2A	113.5 (3)	C3C—C1C—C2C	112.0 (3)
C1A—C2A—H2A1	109.5	C1C—C2C—H2C1	109.5
C1A—C2A—H2A2	109.5	C1C—C2C—H2C2	109.5
H2A1—C2A—H2A2	109.5	H2C1—C2C—H2C2	109.5
C1A—C2A—H2A3	109.5	C1C—C2C—H2C3	109.5
H2A1—C2A—H2A3	109.5	H2C1—C2C—H2C3	109.5
H2A2—C2A—H2A3	109.5	H2C2—C2C—H2C3	109.5
C1A—C3A—H3A1	109.5	C1C—C3C—H3C1	109.5
C1A—C3A—H3A2	109.5	C1C—C3C—H3C2	109.5
H3A1—C3A—H3A2	109.5	H3C1—C3C—H3C2	109.5
C1A—C3A—H3A3	109.5	C1C—C3C—H3C3	109.5
H3A1—C3A—H3A3	109.5	H3C1—C3C—H3C3	109.5
H3A2—C3A—H3A3	109.5	H3C2—C3C—H3C3	109.5
O1A—C4A—C8A	108.4 (2)	O2C—C4C—C6C	112.3 (3)
O1A—C4A—C5A	101.9 (2)	O2C—C4C—C5C	102.5 (2)
C8A—C4A—C5A	118.6 (2)	C6C—C4C—C5C	115.5 (2)
O1A—C4A—H4A	109.2	O2C—C4C—H4C	108.7
C8A—C4A—H4A	109.2	C6C—C4C—H4C	108.7
C5A—C4A—H4A	109.2	C5C—C4C—H4C	108.7
O2A—C5A—C6A	112.8 (2)	O1C—C5C—C8C	109.4 (2)
O2A—C5A—C4A	101.5 (2)	O1C—C5C—C4C	101.8 (2)
C6A—C5A—C4A	115.5 (2)	C8C—C5C—C4C	118.6 (2)
O2A—C5A—H5A	108.9	O1C—C5C—H5C	108.9
C6A—C5A—H5A	108.9	C8C—C5C—H5C	108.9
C4A—C5A—H5A	108.9	C4C—C5C—H5C	108.9
O3A—C6A—C7A	110.7 (2)	O3C—C6C—C7C	110.5 (2)
O3A—C6A—C5A	106.3 (2)	O3C—C6C—C4C	105.4 (2)
C7A—C6A—C5A	111.1 (2)	C7C—C6C—C4C	110.9 (2)
O3A—C6A—H6A	109.6	O3C—C6C—H6C	110.0
C7A—C6A—H6A	109.6	C7C—C6C—H6C	110.0
C5A—C6A—H6A	109.6	C4C—C6C—H6C	110.0
O4A—C7A—C6A	112.8 (3)	O4C—C7C—C6C	112.0 (2)
O4A—C7A—H7A1	109.0	O4C—C7C—H7C1	109.2
C6A—C7A—H7A1	109.0	C6C—C7C—H7C1	109.2
O4A—C7A—H7A2	109.0	O4C—C7C—H7C2	109.2
C6A—C7A—H7A2	109.0	C6C—C7C—H7C2	109.2
H7A1—C7A—H7A2	107.8	H7C1—C7C—H7C2	107.9
N1A—C8A—C4A	109.8 (2)	N1C—C8C—C5C	110.3 (2)
N1A—C8A—H8A1	109.7	N1C—C8C—H8C1	109.6
C4A—C8A—H8A1	109.7	C5C—C8C—H8C1	109.6
N1A—C8A—H8A2	109.7	N1C—C8C—H8C2	109.6
C4A—C8A—H8A2	109.7	C5C—C8C—H8C2	109.6
H8A1—C8A—H8A2	108.2	H8C1—C8C—H8C2	108.1
N2A—C9A—N1A	112.1 (3)	N2C—C9C—N1C	112.5 (3)
N2A—C9A—H9A	123.9	N2C—C9C—H9C	123.8
N1A—C9A—H9A	123.9	N1C—C9C—H9C	123.8
N3A—C10A—N1A	122.9 (3)	N1C—C10C—C11C	105.6 (3)
N3A—C10A—C11A	131.5 (3)	N1C—C10C—N3C	122.9 (2)
N1A—C10A—C11A	105.3 (3)	C11C—C10C—N3C	131.4 (3)
C10A—C11A—N2A	110.4 (2)	C10C—C11C—N2C	110.4 (2)
C10A—C11A—C12A	125.9 (3)	C10C—C11C—C12C	124.4 (3)
N2A—C11A—C12A	123.7 (2)	N2C—C11C—C12C	125.1 (2)
O5A—C12A—N4A	122.7 (3)	O5C—C12C—N4C	123.1 (3)
O5A—C12A—C11A	121.1 (3)	O5C—C12C—C11C	119.7 (2)
N4A—C12A—C11A	116.3 (3)	N4C—C12C—C11C	117.3 (3)
C9A—N1A—C10A	107.1 (2)	C9C—N1C—C10C	106.7 (2)
C9A—N1A—C8A	126.1 (2)	C9C—N1C—C8C	126.9 (2)
C10A—N1A—C8A	126.6 (2)	C10C—N1C—C8C	126.4 (3)
C9A—N2A—C11A	105.1 (2)	C9C—N2C—C11C	104.8 (2)
C10A—N3A—H3NA	112 (3)	C10C—N3C—H3NC	111 (2)
C10A—N3A—H6NA	114 (3)	C10C—N3C—H6NC	110 (2)
H3NA—N3A—H6NA	110 (4)	H3NC—N3C—H6NC	113 (3)
C12A—N4A—H4NA	118 (2)	C12C—N4C—H4NC	122 (2)
C12A—N4A—H5NA	122 (2)	C12C—N4C—H5NC	121 (2)
H4NA—N4A—H5NA	119 (3)	H4NC—N4C—H5NC	116 (3)
C4A—O1A—C1A	106.5 (2)	C5C—O1C—C1C	106.5 (2)
C5A—O2A—C1A	109.6 (2)	C4C—O2C—C1C	110.7 (2)
C6A—O3A—H3AO	109 (3)	C6C—O3C—H3CO	111 (3)
C7A—O4A—H4AO	106 (3)	C7C—O4C—H4CO	103 (3)
O2B—C1B—O1B	104.3 (2)	O1D—C1D—O2D	105.1 (2)
O2B—C1B—C3B	110.2 (3)	O1D—C1D—C3D	108.0 (3)
O1B—C1B—C3B	108.7 (3)	O2D—C1D—C3D	109.8 (3)
O2B—C1B—C2B	109.4 (3)	O1D—C1D—C2D	112.0 (3)
O1B—C1B—C2B	111.5 (3)	O2D—C1D—C2D	108.5 (3)
C3B—C1B—C2B	112.5 (3)	C3D—C1D—C2D	113.2 (3)
C1B—C2B—H2B1	109.5	C1D—C2D—H2D1	109.5
C1B—C2B—H2B2	109.5	C1D—C2D—H2D2	109.5
H2B1—C2B—H2B2	109.5	H2D1—C2D—H2D2	109.5
C1B—C2B—H2B3	109.5	C1D—C2D—H2D3	109.5
H2B1—C2B—H2B3	109.5	H2D1—C2D—H2D3	109.5
H2B2—C2B—H2B3	109.5	H2D2—C2D—H2D3	109.5
C1B—C3B—H3B1	109.5	C1D—C3D—H3D1	109.5
C1B—C3B—H3B2	109.5	C1D—C3D—H3D2	109.5
H3B1—C3B—H3B2	109.5	H3D1—C3D—H3D2	109.5
C1B—C3B—H3B3	109.5	C1D—C3D—H3D3	109.5
H3B1—C3B—H3B3	109.5	H3D1—C3D—H3D3	109.5
H3B2—C3B—H3B3	109.5	H3D2—C3D—H3D3	109.5
O2B—C4B—C6B	112.2 (3)	O2D—C4D—C6D	113.1 (2)
O2B—C4B—C5B	103.1 (2)	O2D—C4D—C5D	101.5 (2)
C6B—C4B—C5B	115.4 (2)	C6D—C4D—C5D	114.9 (2)
O2B—C4B—H4B	108.6	O2D—C4D—H4D	109.0
C6B—C4B—H4B	108.6	C6D—C4D—H4D	109.0
C5B—C4B—H4B	108.6	C5D—C4D—H4D	109.0
O1B—C5B—C8B	108.9 (2)	O1D—C5D—C8D	108.1 (2)
O1B—C5B—C4B	101.9 (2)	O1D—C5D—C4D	101.7 (2)
C8B—C5B—C4B	118.0 (2)	C8D—C5D—C4D	118.9 (2)
O1B—C5B—H5B	109.2	O1D—C5D—H5D	109.2
C8B—C5B—H5B	109.2	C8D—C5D—H5D	109.2
C4B—C5B—H5B	109.2	C4D—C5D—H5D	109.2
O3B—C6B—C7B	111.0 (2)	O3D—C6D—C7D	111.3 (2)
O3B—C6B—C4B	105.0 (2)	O3D—C6D—C4D	105.6 (2)
C7B—C6B—C4B	112.4 (2)	C7D—C6D—C4D	112.0 (2)
O3B—C6B—H6B	109.4	O3D—C6D—H6D	109.3
C7B—C6B—H6B	109.4	C7D—C6D—H6D	109.3
C4B—C6B—H6B	109.4	C4D—C6D—H6D	109.3
O4B—C7B—C6B	111.1 (2)	O4D—C7D—C6D	111.8 (3)
O4B—C7B—H7B1	109.4	O4D—C7D—H7D1	109.3
C6B—C7B—H7B1	109.4	C6D—C7D—H7D1	109.3
O4B—C7B—H7B2	109.4	O4D—C7D—H7D2	109.3
C6B—C7B—H7B2	109.4	C6D—C7D—H7D2	109.3
H7B1—C7B—H7B2	108.0	H7D1—C7D—H7D2	107.9
N1B—C8B—C5B	110.9 (2)	N1D—C8D—C5D	109.8 (2)
N1B—C8B—H8B1	109.5	N1D—C8D—H8D1	109.7
C5B—C8B—H8B1	109.5	C5D—C8D—H8D1	109.7
N1B—C8B—H8B2	109.5	N1D—C8D—H8D2	109.7
C5B—C8B—H8B2	109.5	C5D—C8D—H8D2	109.7
H8B1—C8B—H8B2	108.0	H8D1—C8D—H8D2	108.2
N2B—C9B—N1B	112.5 (3)	N2D—C9D—N1D	112.4 (3)
N2B—C9B—H9B	123.7	N2D—C9D—H9D	123.8
N1B—C9B—H9B	123.7	N1D—C9D—H9D	123.8
N3B—C10B—N1B	122.8 (2)	N3D—C10D—N1D	122.8 (3)
N3B—C10B—C11B	132.5 (3)	N3D—C10D—C11D	131.5 (3)
N1B—C10B—C11B	104.7 (3)	N1D—C10D—C11D	105.4 (3)
C10B—C11B—N2B	110.6 (2)	C10D—C11D—N2D	110.1 (2)
C10B—C11B—C12B	126.2 (3)	C10D—C11D—C12D	126.4 (3)
N2B—C11B—C12B	123.1 (2)	N2D—C11D—C12D	123.3 (2)
O5B—C12B—N4B	122.3 (2)	O5D—C12D—N4D	123.3 (3)
O5B—C12B—C11B	120.3 (2)	O5D—C12D—C11D	121.0 (2)
N4B—C12B—C11B	117.4 (3)	N4D—C12D—C11D	115.7 (3)
C9B—N1B—C10B	107.6 (2)	C9D—N1D—C10D	107.1 (2)
C9B—N1B—C8B	126.1 (2)	C9D—N1D—C8D	126.1 (2)
C10B—N1B—C8B	126.3 (3)	C10D—N1D—C8D	126.7 (2)
C9B—N2B—C11B	104.5 (2)	C9D—N2D—C11D	104.9 (2)
C10B—N3B—H3NB	114 (2)	C10D—N3D—H3ND	114 (3)
C10B—N3B—H6NB	115 (2)	C10D—N3D—H6ND	115 (2)
H3NB—N3B—H6NB	110 (3)	H3ND—N3D—H6ND	108 (3)
C12B—N4B—H4NB	116 (2)	C12D—N4D—H4ND	122 (2)
C12B—N4B—H5NB	122 (2)	C12D—N4D—H5ND	118 (2)
H4NB—N4B—H5NB	122 (3)	H4ND—N4D—H5ND	117 (3)
C5B—O1B—C1B	106.8 (2)	C5D—O1D—C1D	106.2 (2)
C4B—O2B—C1B	110.8 (2)	C4D—O2D—C1D	109.9 (2)
C6B—O3B—H3BO	113 (2)	C6D—O3D—H3DO	106 (2)
C7B—O4B—H4BO	105 (2)	C7D—O4D—H4DO	109 (3)
			
O1A—C4A—C5A—O2A	36.6 (3)	O2C—C4C—C5C—O1C	30.6 (3)
C8A—C4A—C5A—O2A	155.4 (3)	C6C—C4C—C5C—O1C	−91.8 (3)
O1A—C4A—C5A—C6A	−85.8 (3)	O2C—C4C—C5C—C8C	150.6 (3)
C8A—C4A—C5A—C6A	33.1 (4)	C6C—C4C—C5C—C8C	28.2 (4)
O2A—C5A—C6A—O3A	164.3 (2)	O2C—C4C—C6C—O3C	165.3 (2)
C4A—C5A—C6A—O3A	−79.6 (3)	C5C—C4C—C6C—O3C	−77.6 (3)
O2A—C5A—C6A—C7A	43.9 (3)	O2C—C4C—C6C—C7C	45.7 (3)
C4A—C5A—C6A—C7A	159.9 (2)	C5C—C4C—C6C—C7C	162.8 (3)
O3A—C6A—C7A—O4A	59.0 (3)	O3C—C6C—C7C—O4C	62.4 (3)
C5A—C6A—C7A—O4A	176.8 (2)	C4C—C6C—C7C—O4C	178.8 (2)
O1A—C4A—C8A—N1A	−57.2 (3)	O1C—C5C—C8C—N1C	−58.9 (3)
C5A—C4A—C8A—N1A	−172.6 (2)	C4C—C5C—C8C—N1C	−174.9 (3)
N3A—C10A—C11A—N2A	174.9 (3)	N1C—C10C—C11C—N2C	0.2 (3)
N1A—C10A—C11A—N2A	0.7 (3)	N3C—C10C—C11C—N2C	175.3 (3)
N3A—C10A—C11A—C12A	−5.3 (5)	N1C—C10C—C11C—C12C	178.4 (3)
N1A—C10A—C11A—C12A	−179.6 (3)	N3C—C10C—C11C—C12C	−6.5 (5)
C10A—C11A—C12A—O5A	5.1 (5)	C10C—C11C—C12C—O5C	6.6 (4)
N2A—C11A—C12A—O5A	−175.2 (3)	N2C—C11C—C12C—O5C	−175.5 (3)
C10A—C11A—C12A—N4A	−173.3 (3)	C10C—C11C—C12C—N4C	−172.7 (3)
N2A—C11A—C12A—N4A	6.3 (4)	N2C—C11C—C12C—N4C	5.2 (4)
N2A—C9A—N1A—C10A	1.1 (4)	N2C—C9C—N1C—C10C	0.5 (3)
N2A—C9A—N1A—C8A	175.9 (3)	N2C—C9C—N1C—C8C	178.1 (3)
N3A—C10A—N1A—C9A	−175.9 (3)	C11C—C10C—N1C—C9C	−0.4 (3)
C11A—C10A—N1A—C9A	−1.0 (3)	N3C—C10C—N1C—C9C	−176.1 (3)
N3A—C10A—N1A—C8A	9.3 (5)	C11C—C10C—N1C—C8C	−178.0 (3)
C11A—C10A—N1A—C8A	−175.8 (3)	N3C—C10C—N1C—C8C	6.3 (4)
C4A—C8A—N1A—C9A	−80.1 (3)	C5C—C8C—N1C—C9C	−86.2 (3)
C4A—C8A—N1A—C10A	93.7 (4)	C5C—C8C—N1C—C10C	90.9 (3)
N1A—C9A—N2A—C11A	−0.6 (3)	N1C—C9C—N2C—C11C	−0.4 (3)
C10A—C11A—N2A—C9A	−0.1 (3)	C10C—C11C—N2C—C9C	0.1 (3)
C12A—C11A—N2A—C9A	−179.8 (3)	C12C—C11C—N2C—C9C	−178.1 (3)
C8A—C4A—O1A—C1A	−163.8 (2)	C8C—C5C—O1C—C1C	−163.9 (2)
C5A—C4A—O1A—C1A	−38.0 (3)	C4C—C5C—O1C—C1C	−37.5 (3)
O2A—C1A—O1A—C4A	24.6 (3)	O2C—C1C—O1C—C5C	30.0 (3)
C3A—C1A—O1A—C4A	142.0 (3)	C3C—C1C—O1C—C5C	147.5 (3)
C2A—C1A—O1A—C4A	−92.8 (3)	C2C—C1C—O1C—C5C	−88.2 (3)
C6A—C5A—O2A—C1A	101.6 (3)	C6C—C4C—O2C—C1C	111.3 (3)
C4A—C5A—O2A—C1A	−22.5 (3)	C5C—C4C—O2C—C1C	−13.3 (3)
O1A—C1A—O2A—C5A	0.2 (3)	O1C—C1C—O2C—C4C	−9.1 (3)
C3A—C1A—O2A—C5A	−115.7 (3)	C3C—C1C—O2C—C4C	−126.0 (3)
C2A—C1A—O2A—C5A	119.4 (3)	C2C—C1C—O2C—C4C	110.7 (3)
O2B—C4B—C5B—O1B	27.9 (3)	O2D—C4D—C5D—O1D	36.2 (3)
C6B—C4B—C5B—O1B	−94.8 (3)	C6D—C4D—C5D—O1D	−86.2 (3)
O2B—C4B—C5B—C8B	147.1 (3)	O2D—C4D—C5D—C8D	154.7 (3)
C6B—C4B—C5B—C8B	24.5 (4)	C6D—C4D—C5D—C8D	32.2 (3)
O2B—C4B—C6B—O3B	164.1 (2)	O2D—C4D—C6D—O3D	163.0 (2)
C5B—C4B—C6B—O3B	−78.2 (3)	C5D—C4D—C6D—O3D	−81.0 (3)
O2B—C4B—C6B—C7B	43.3 (3)	O2D—C4D—C6D—C7D	41.7 (3)
C5B—C4B—C6B—C7B	161.0 (3)	C5D—C4D—C6D—C7D	157.7 (2)
O3B—C6B—C7B—O4B	63.3 (3)	O3D—C6D—C7D—O4D	59.2 (3)
C4B—C6B—C7B—O4B	−179.4 (2)	C4D—C6D—C7D—O4D	177.2 (2)
O1B—C5B—C8B—N1B	−57.6 (3)	O1D—C5D—C8D—N1D	−57.0 (3)
C4B—C5B—C8B—N1B	−173.0 (3)	C4D—C5D—C8D—N1D	−172.0 (2)
N3B—C10B—C11B—N2B	−179.7 (3)	N3D—C10D—C11D—N2D	174.9 (3)
N1B—C10B—C11B—N2B	1.3 (3)	N1D—C10D—C11D—N2D	0.9 (3)
N3B—C10B—C11B—C12B	−3.3 (5)	N3D—C10D—C11D—C12D	−9.4 (5)
N1B—C10B—C11B—C12B	177.6 (3)	N1D—C10D—C11D—C12D	176.6 (3)
C10B—C11B—C12B—O5B	14.5 (4)	C10D—C11D—C12D—O5D	8.8 (5)
N2B—C11B—C12B—O5B	−169.5 (3)	N2D—C11D—C12D—O5D	−176.1 (3)
C10B—C11B—C12B—N4B	−164.9 (3)	C10D—C11D—C12D—N4D	−169.1 (3)
N2B—C11B—C12B—N4B	11.1 (4)	N2D—C11D—C12D—N4D	6.0 (4)
N2B—C9B—N1B—C10B	0.5 (4)	N2D—C9D—N1D—C10D	0.4 (4)
N2B—C9B—N1B—C8B	−179.9 (3)	N2D—C9D—N1D—C8D	177.8 (3)
N3B—C10B—N1B—C9B	179.8 (3)	N3D—C10D—N1D—C9D	−175.5 (3)
C11B—C10B—N1B—C9B	−1.1 (3)	C11D—C10D—N1D—C9D	−0.8 (3)
N3B—C10B—N1B—C8B	0.2 (4)	N3D—C10D—N1D—C8D	7.2 (5)
C11B—C10B—N1B—C8B	179.4 (3)	C11D—C10D—N1D—C8D	−178.1 (3)
C5B—C8B—N1B—C9B	−89.4 (3)	C5D—C8D—N1D—C9D	−80.6 (3)
C5B—C8B—N1B—C10B	90.1 (3)	C5D—C8D—N1D—C10D	96.2 (3)
N1B—C9B—N2B—C11B	0.3 (3)	N1D—C9D—N2D—C11D	0.1 (3)
C10B—C11B—N2B—C9B	−1.0 (3)	C10D—C11D—N2D—C9D	−0.6 (3)
C12B—C11B—N2B—C9B	−177.5 (3)	C12D—C11D—N2D—C9D	−176.5 (3)
C8B—C5B—O1B—C1B	−162.2 (2)	C8D—C5D—O1D—C1D	−164.5 (2)
C4B—C5B—O1B—C1B	−36.8 (3)	C4D—C5D—O1D—C1D	−38.6 (3)
O2B—C1B—O1B—C5B	31.6 (3)	O2D—C1D—O1D—C5D	26.1 (3)
C3B—C1B—O1B—C5B	149.1 (3)	C3D—C1D—O1D—C5D	143.3 (3)
C2B—C1B—O1B—C5B	−86.3 (3)	C2D—C1D—O1D—C5D	−91.5 (3)
C6B—C4B—O2B—C1B	115.2 (3)	C6D—C4D—O2D—C1D	102.3 (3)
C5B—C4B—O2B—C1B	−9.6 (3)	C5D—C4D—O2D—C1D	−21.4 (3)
O1B—C1B—O2B—C4B	−12.5 (3)	O1D—C1D—O2D—C4D	−1.5 (3)
C3B—C1B—O2B—C4B	−129.0 (3)	C3D—C1D—O2D—C4D	−117.4 (3)
C2B—C1B—O2B—C4B	106.9 (3)	C2D—C1D—O2D—C4D	118.5 (3)

### Hydrogen-bond geometry (Å, º)

**Table:**

*D*—H···*A*	*D*—H	H···*A*	*D*···*A*	*D*—H···*A*
C8*A*—H8*A*1···O4*C*^i^	0.97	2.62	3.444 (4)	143
N3*A*—H3*NA*···O5*A*	0.89 (4)	2.32 (4)	2.945 (4)	127 (3)
N3*A*—H6*NA*···O1*A*	0.89 (4)	2.66 (4)	3.250 (4)	125 (3)
N4*A*—H4*NA*···O3*A*^ii^	0.92 (4)	2.05 (4)	2.973 (3)	178 (3)
N4*A*—H5*NA*···N2*C*	0.91 (4)	2.23 (4)	3.064 (4)	153 (3)
O3*A*—H3*AO*···O4*C*^i^	0.79 (4)	2.05 (4)	2.831 (3)	170 (4)
O4*A*—H4*AO*···O5*A*^iii^	0.80 (4)	2.01 (4)	2.798 (3)	166 (4)
C3*B*—H3*B*1···N1*D*^iv^	0.96	2.58	3.398 (5)	144
C8*B*—H8*B*1···O5*C*^v^	0.97	2.51	3.243 (4)	132
C8*B*—H8*B*2···O4*A*^vi^	0.97	2.43	3.291 (4)	147
N3*B*—H3*NB*···O1*B*	0.89 (3)	2.35 (3)	3.037 (3)	133 (3)
N3*B*—H6*NB*···O5*B*	0.95 (4)	2.39 (3)	2.977 (3)	120 (3)
N3*B*—H6*NB*···O5*D*^iv^	0.95 (4)	2.52 (4)	3.196 (4)	129 (3)
N4*B*—H4*NB*···O3*B*^iii^	0.89 (4)	2.03 (4)	2.901 (3)	170 (3)
N4*B*—H5*NB*···N2*D*^iii^	0.85 (4)	2.16 (4)	2.920 (4)	150 (3)
O3*B*—H3*BO*···N3*C*^v^	0.87 (3)	1.98 (4)	2.838 (4)	168 (3)
O4*B*—H4*BO*···O5*B*^ii^	0.91 (4)	1.81 (4)	2.715 (3)	170 (3)
C8*C*—H8*C*1···O5*B*^vii^	0.97	2.64	3.380 (3)	133
C8*C*—H8*C*2···O4*D*^i^	0.97	2.41	3.360 (4)	166
N3*C*—H3*NC*···O1*C*	0.87 (4)	2.48 (4)	3.152 (4)	134 (3)
N3*C*—H6*NC*···O5*C*	0.90 (4)	2.21 (3)	2.874 (3)	130 (3)
N4*C*—H4*NC*···N2*A*	0.86 (4)	2.14 (4)	2.934 (4)	154 (3)
N4*C*—H5*NC*···O3*C*^iii^	0.84 (4)	2.12 (4)	2.959 (3)	176 (4)
O3*C*—H3*CO*···O5*B*^vii^	0.80 (4)	2.03 (4)	2.823 (3)	173 (3)
O4*C*—H4*CO*···O5*C*^ii^	0.80 (4)	1.97 (4)	2.738 (3)	162 (4)
N3*D*—H6*ND*···N4*C*^viii^	0.92 (4)	2.62 (4)	3.268 (4)	128 (3)
N3*D*—H6*ND*···O5*D*	0.92 (4)	2.36 (4)	2.972 (4)	124 (3)
N4*D*—H4*ND*···N2*B*^ii^	0.89 (4)	2.26 (4)	3.075 (4)	153 (3)
N4*D*—H5*ND*···O3*D*^ii^	0.87 (4)	2.09 (4)	2.957 (3)	176 (3)
O3*D*—H3*DO*···O4*B*^ix^	0.90 (4)	1.82 (4)	2.711 (3)	171 (3)
O4*D*—H4*DO*···O5*D*^iii^	0.79 (3)	2.06 (3)	2.822 (3)	164 (4)

Symmetry codes: (i) −*x*+2, *y*−1/2, −*z*+1; (ii) *x*+1, *y*, *z*; (iii) *x*−1, *y*, *z*; (iv) −*x*+2, *y*−1/2, −*z*+2; (v) *x*, *y*, *z*+1; (vi) −*x*+1, *y*+1/2, −*z*+2; (vii) *x*+1, *y*, *z*−1; (viii) −*x*+2, *y*+1/2, −*z*+1; (ix) −*x*+2, *y*+1/2, −*z*+2.
